# Toward Exploring Novel Organic Materials: MP4-DFT Properties of 4-Amino-3-Iminoindene

**DOI:** 10.3390/molecules22050720

**Published:** 2017-04-30

**Authors:** Tareq Irshaidat

**Affiliations:** Department of Chemistry, College of Sciences, Al-Hussein Bin Talal University, P.O. Box 20, Ma’an, Jordan; tirshaidat@yahoo.com; Tel.: +962-799-514-989

**Keywords:** tautomerism, MP4(SDTQ), molecular switching, CAM-B3LYP, frontier orbitals, semiconductor

## Abstract

Tautomerism links with many applications and remains an attracting feature in exploring novel systems. In this regard, properties of indene-based HNCCCN segments have not received any considerable attention. In this computational organic chemistry study, first, to calculate the proton transfer energy barrier at a reasonable cost, the study identified an accurate forth order Møller–Plesset perturbation theory-density functional theory (MP4-DFT) protocol equivalent to the outstanding pioneering benchmark calculations. The calculations illustrate that the two tautomers of the 4-amino-3-iminoindene nucleus are separated by a considerable energy barrier while featuring different molecular orbital characteristics; frontier orbital distribution, λmax, and energies, which are known basic requirements in molecular switching and logic circuit applications. The N-H/BH_2_ substitution was found to have significant influence on the electronic structure of the skeleton. Similarities in the two tautomers and the boron derivative to properties of known molecular materials have been found.

## 1. Introduction

**Figure 1:**
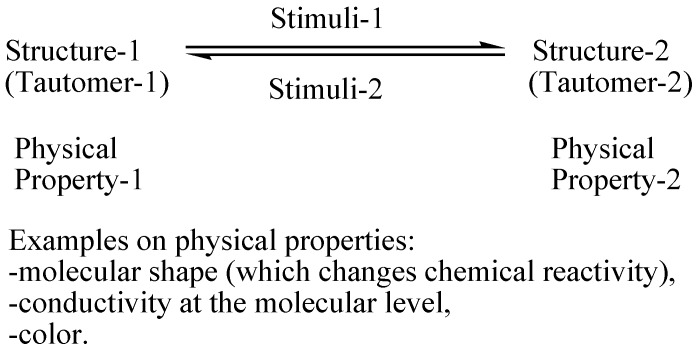
A simplified illustration for the concept of “molecular switching”.

Tautomerism is a fundamental concept in organic chemistry and an interesting phenomenon as it associates with many important chemical and biological processes [1]. When tautomers exhibit different physical properties, it is possible, in principle, to invest this in molecular switching applications. However, in many cases the barrier between the tautomers may be low and, therefore, the tautomers exist together in the medium. In such cases, structure modification is necessary to control the formation of one tautomer at a time to recognize the switching process (on/off of the desired physical properties) (Figure 1) [2]. Among the promising applications is the molecular computer and all of the accompanying advantages of miniaturizing the hardware.

Switching based on tautomerization reactions has been recognized in many molecular systems [3,4,5,6,7,8,9,10]. The first recognized ideal tautomerism-based molecular switching process was identified for the porphyrins-category of molecules [11]. The proton transfers from one nitrogen atom to another can take place in a controllable manner by applying a voltage at low temperature using a scanning tunneling microscope (STM). Switching between the tautomers causes a change in the conductivity from one level to another. Later on, another example of the switching between desired tautomers was achieved, leading to a multi-control of the molecular conductivity [12]. Another type of promising tautomerism-based molecular switching is based on the quinone core. Nitrogen derivatives were studied on the surface of Cu(110) as a supporting platform. The study confirms that the molecular conductivity can be controlled in a similar way using the STM [13].

**Figure 2:**
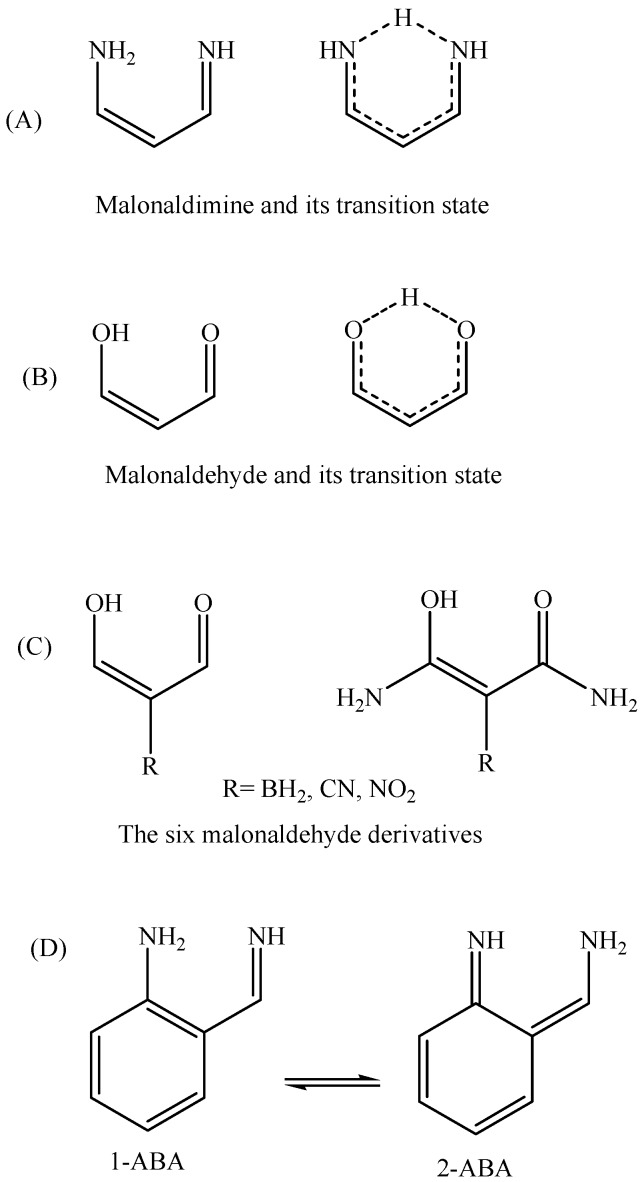
Structures of malonaldimine (**A**); malonaldehyde (**B**) and its derivatives (**C**); and 2-aminobenzaldimine tautomers (**D**).

The basic requirement to observe one dominant (main) tautomer is high activation energy for the proton transfer process. In a previous study [14] focused on characterizing malonaldimine (NCCCN skeleton, Figure 2A) as a potential core for asymmetrical molecular switching, we found that the proton transfer energy barrier is, nearly, 9 kcal/mol. In order to increase the selectivity among asymmetrical tautomers (containing the NCCCN core) it is necessary to push the activation energy to a higher value. As can be seen in the previous studies [3,4,5,6,7,8,9,10], aromaticity can be employed here to generate asymmetrical tautomers with different electronic structures; one is aromatic and the other is not aromatic. Additionally, introducing a skeletal constraint that guarantees pushing the two nitrogen atoms further apart from each other (compared to malonaldimine; N…N = 2.7088 Å) can participate in making the proton transfer more difficult and, thus, energetically more expensive. After initial evaluations, we found that indene skeleton is a reasonable candidate. Attaching amine and imine groups in a way that guarantees the transfer of protons and the subsequent formation of two tautomers is possible in many ways. 

**Figure 3:**
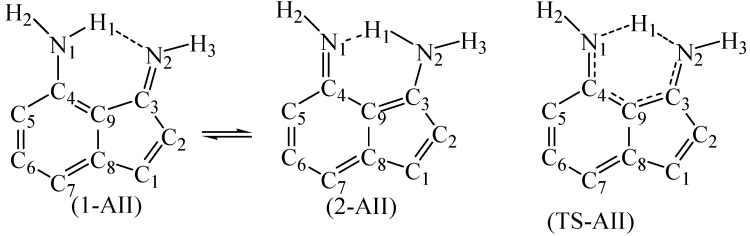
The numbering system of the two tautomers (1-AII and 2-AII) and the transition state (TS-AII) of (4-Amino-3-imino)indene. The other hydrogen atoms are omitted for simplicity.

**Figure 4:**
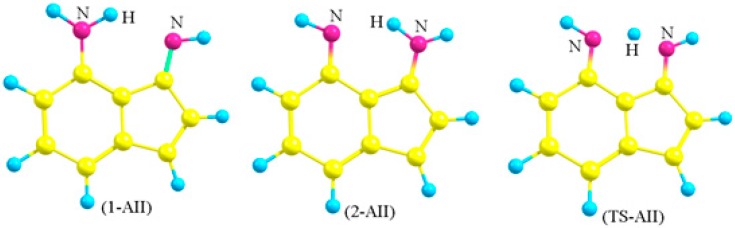
The optimized structures of the two tautomers (1-AII and 2-AII) and the transition state (TS-AII) of (4-Amino-3-imino)indene.

Choosing 4-amino-3-iminoindene was inspired from astonishing contemporary syntheses. Recently, the indispensable review by Gabriele and co-workers presented many new methods for synthesis of indene derivatives [15]. Among them, Tsukamoto and co-workers [16] showed that it is possible to produce 3-aminoindene derivatives starting from alkynylbenzaldehyde, secondary amine, R-B(OH)_2_, and a palladium catalyst. On the other hand, Liu and co-workers [17] presented another successful example on producing similar derivatives, but used a different strategy that depends on the coupling of iodobenzaldimine and alkynes in the presence of zinc and a cobalt catalyst. Starting with a protected amine group on the benzene ring of the starting material (alkynylbenzaldehyde or iodobenzaldimine), followed by the indene-forming reaction, oxidation, then deprotection of the amine group would produce derivatives of amino-3-iminoindene. The simplest form of the 4-amino-3-iminoindene nucleus was adopted for this study. The amine group, being on C4, allows this structure to exist in two asymmetrical tautomeric forms (1-AII and 2-AII; Figure 3 and Figure 4) separated by the transition state (TS-AII). The purpose of this study is to explore the electronic structure features of 4-amino-3-iminoindene. The remainder of this text is arranged as follows: the computational details section, selecting a reasonable MP4(SDTQ) protocol to calculate the proton transfer activation energy section, and the frontier orbitals and the optical properties section.

## 2. Computational Methods

As recommended earlier [18], the Becke, three-parameter, Lee-Yang-Parr exchange-correlation functional B3LYP [19,20] and the 6-31G(d,p) basis set [21] were used to optimize all of the geometries. These calculations gave real frequencies for 1-AII and 2-AII, which indicates that each is a true minimum. On the other hand, the calculation gave one imaginary frequency for TS-AII associated with the vibration of the hydrogen between the two nitrogen atoms, which confirms the identity of the transition state. The forth order Møller–Plesset perturbation theory; MP4(SDTQ)/6-31G(d,p) [22], calculations were performed to obtain single-point energies. The Gaussian03 (Revision E 0.1; Gaussian, Inc.: Pittsburgh, PA, USA) suite of programs was used to perform these calculations [23]. The time-dependent (TD) calculations [24,25,26] were performed to estimate the vertical excitation energy using thecoulomb-attenuating method of the B3LYP functional CAM-B3LYP [27] along with the 6-31G(d) basis set, as implemented in GAMESS suite of programs [28,29] (Gamess is maintained by the members of the Gordon Research Group at Iowa State University, Ames, Iowa, USA).

## 3. Results and Discussion

### 3.1. MP4(SDTQ)/B3LYP Protocol to Calculate the Proton Transfer Activation Energy

The proton transfer activation energy of malonaldehyde (Figure 2B) was calculated using the coupled-cluster with single and double excitations and an approximate treatment of triples at the complete basis set; CCSD(T)/CBS, level of theory and found to be equal to 4.09 kcal/mol [30]. The availability of this value allows searching for less demanding alternative methods. Previously, we found that both B3LYP/6-31++G(3df,3pd) and MP2/6-31++G(3df,3pd) protocols fail in predicting the proton transfer activation energy of malonaldehyde [18]. Table 1 presents the results of various basis set types along with the MP4(SDTQ) method. The reason behind avoiding larger basis sets is to keep the calculations at as low a price as possible. Among all of the basis sets 6-31G(d,p) shows the best performance (|∆∆Eb| = 0.02 kcal/mol) in terms of accuracy and the computation time. 

**Figure 5:**
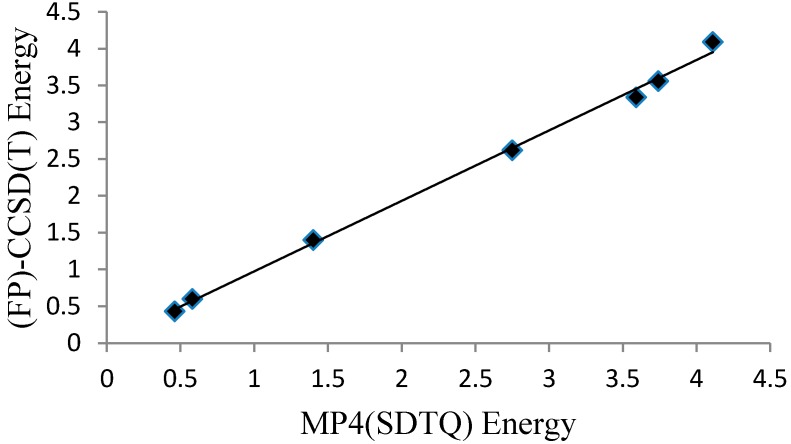
A graph presenting a linear relationship between the MP4(SDTQ) and (FP)-CCSD(T) energies.

The MP4(SDTQ)/6-31G(d,p) level of theory value is equal to 4.11 kcal/mol, which is an excellent estimation compared to the more demanding CCSD(T) [18] and the quadratic configuration interaction with single and double excitations and an approximation of the triples; QCISD(T) [14], protocols. Further evaluation of the MP4(SDTQ)/6-31G(d,p)//B3LYP/6-31G(d,p) protocol was performed on six malonaldehyde derivatives (Figure 2C, Table 2) where the reference values are focal point (FP) CCSD(T) energies [31]. The average deviation from the accurate focal point values (|∆∆Eb|) are 0.19 and 0.02 kcal/mol for the two groups of malonaldehyde derivatives. Plotting the MP4(SDTQ) energies against the (FP)-CCSD(T) energies of the six derivatives, in addition to the malonaldehyde value (Figure 5), can produce a simple linear relationship between the two with high precision. Therefore, the results confirm that the MP4(SDTQ) protocol is in excellent agreement with the accurate FP-CCSD(T) protocol and may be considered a reasonable protocol to estimate the energy barrier of the proton transfer process.

In light of these results, the energy barrier of AII (1-AII → TS-AII, Table 2) was calculated using the MP4(SDTQ)/6-31G(d,p)//B3LYP protocol. As a consequence, the activation energy of 1-AII (MP4 = 22.41 kcal/mol compared to MP4 = 9.15 kcal/mol in malonaldimine) indicates that the transformation from 1-AII to 2-AII is not spontaneous, yet kinetically possible. On the other hand, going in the opposite direction (2-AII to TS-AII) requires lower energy (5.72 kcal/mol) and the value indicates that the absence of the first external stimuli (stimuli-1) allows retrieving the first tautomer (1-AII) by a smaller energy (stimuli-2). The nature of stimuli-2 depends on the operating temperature. A support to the values of the proton transfer activation energies comes from the CCSD(T)/D95(d,p)//B3LYP/6-31G(d,p) and QCISD(T)/D95(d,p)//B3LYP/6-31G(d,p) protocols, as recommended earlier [14,18]. In a previous work, we demonstrated that, for a specific molecular system, tautomerism is a controllable process [32]. Therefore, fine-tuning of the barriers (increasing/decreasing) and the relative energies of tautomers may be achieved by a suitable substituent. 

In order to obtain a qualitative idea about the contribution of the aromaticity and the N…N distance in increasing the transition state energy the tautomerization reaction of 2-aminobenzaldimine (2-NH_2_-PhCH=NH, Figure 2; 1-ABA and 2-ABA) is compared, in addition to malonaldimine. The N…N distance of 1-ABA is equal to 2.7122 Å, which is a negligible increase (0.0034 Å) compared to that of malonaldimine. The transition state energy, with respect to 1-ABA, was calculated using the MP4(SDTQ) protocol and found to be equal to 16.51 kcal/mol. This increase, with respect to malonaldimine (7.36 kcal/mol), is attributed mainly to the loss of the aromatic stabilization energy. On the other hand, the N…N distance in 1-AII is equal to 2.9326 Å; a 0.2238 Å increase with respect to malonaldimine, and accompanied by an increase in the transition state energy to 22.41 kcal/mol. This indicates 13.26 kcal/mol more than malonaldimine. As a rough estimation (1) 7.36 kcal/mol is due to the loss of the benzene ring aromaticity in AII as a result of the tautomerization reaction; and (2) 5.9 kcal/mol is due to the larger N…N distance (2.9326 Å in 1-AII). Therefore, these observations indicate that, in addition to aromaticity, the N…N distance is an important factor in determining the proton transfer energy barrier.

### 3.2. The Frontier Orbitals of the Two Tautomers

Converting one tautomer to another can be useful only if it is accompanied with a significant change in a physical property. Focusing on the optical property of the tautomers, the initial evaluation of the frontier orbitals—the highest occupied molecular orbital (HOMO) and the lowest unoccupied molecular orbital (LUMO)—illustrated that the HOMO-LUMO energy difference changes from 1-AII to 2-AII. In order to obtain a more quantitative idea about the gaps the TD-DFT theoretical approach was adopted to evaluate the first vertical π → π* excitation energies using the CAM-B3LYP functional along with the 6-31G(d) basis set.

The first vertical π → π* excitation energies of 1-AII and 2-AII were calculated using the TD-(CAM-B3LYP/6-31G(d))//B3LYP/6-31G(d,p) protocol (Table 3) in vacuum, acetonitrile, and water. The results show that the dielectric constant of the medium has only a negligible effect on the energies of the frontier orbitals. This is consistent with the small dipole moment values of the two tautomers (MP4(SDTQ)/6-31G(d,p): 1-AII = 1.255, 2-AII = 2.509 Debye). The calculations in vacuum illustrate that using a larger basis set than 6-31G(d,p) (compared with the triple-zeta TZVP basis set) with the CAM-B3LYP functional is not necessary to obtain reasonable values. 

The ∆λ values (λ(2-AII)-λ(1-AII); Table 3) illustrate that the excitation energies of the two tautomers differ by more than 140 nm and this difference varies slightly by the medium’s dielectric constant. The ∆E values indicate that in 2-AII the valence electron density is less tightly bound to the nuclear charges and can move or transfer easier than in 1-AII. Consequently, and at the molecular level, the molecular conductivity of 2-AII is better. It is obvious (Table 3) that the two tautomers exhibit valuable physical variations (∆∆E ≥ 0.97 (eV) and ∆λ > 140 (nm)) and differ significantly in their relative energies and, as a consequence, supports that derivatives of AII are promising building blocks for molecular switching and signaling applications.

**Figure 6:**
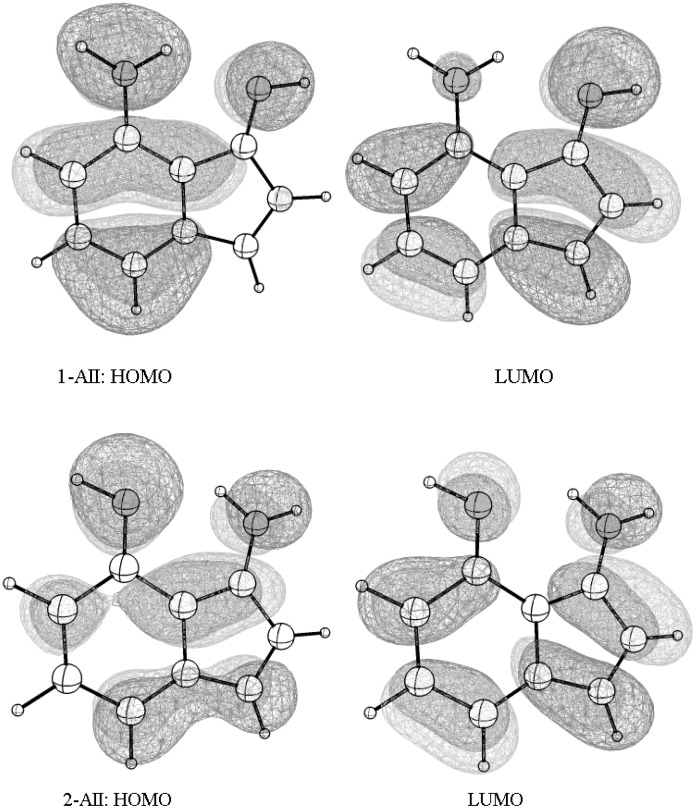
The frontier orbitals—the highest occupied molecular orbital (HOMO) and the lowest unoccupied molecular orbital (LUMO)—spreading over the entire molecular skeleton allow an efficient π-π interaction and intra-molecular electrons flow in the two tautomerism states.

Anthracene, tetracene, and pentacene are known organic semiconductors [33]. The estimated vertical π → π* excitation energy based on TD-(CAM-B3LYP/6-31G(d)) calculations (Table 4) are equal to 3.63 eV, 2.85 eV, and 2.37 eV, which are very close to the experimental values (3.60, 2.88, and 2.37 eV, respectively) [34]. The data gives a clearer qualitative look at the difference in the conductivity at the molecular level and indicate that both tautomers may conduct current. Taking into consideration the frontier orbitals of the two tautomers (Figure 6; 1-AII and 2-AII), they distribute over the entire molecular skeleton (Figure 6) which allows efficient inter-molecular π-π interaction. Relatively, this supports that AII (1-AII ↔ 2-AII) is a new molecular semiconductor candidate. 

The literature shows that both tetracene [35] and pentacene [36] are efficient electron donors when coupled with C60 (as an acceptor) and can be used to fabricate organic solar cells. It is noticed (Table 4) that the frontier orbitals’ energies of 1-AII are close to that of tetracene. The energy of the HOMO orbital (of 1-AII) may be increased by incorporating an electron donating group. However, relatively, the energy of the HOMO orbital at this level, being lower than the HOMO orbital of tetracene, indicates that it is more resistant to the environmental chemical factors and, therefore, gives it the advantage of easier handling and processing.

### 3.3. Effect of N-H/BH_2_ Substitution

**Figure 7:**
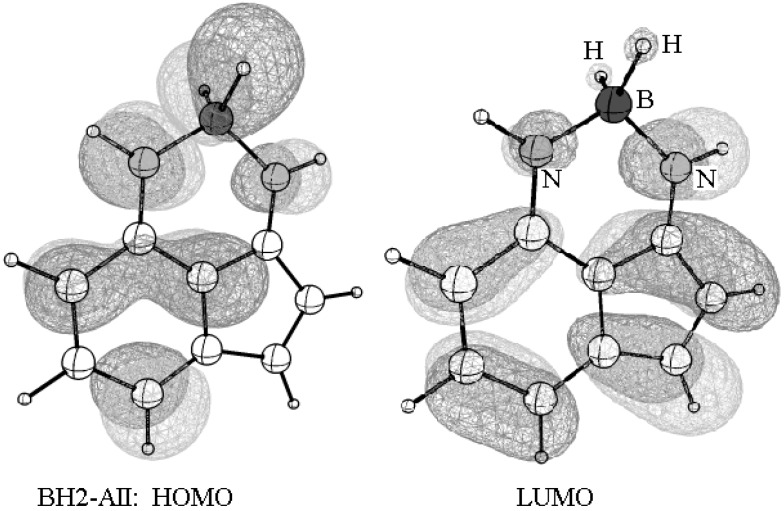
A pictorial representation of the frontier orbitals of BH_2_-AII.

The advantage of the resonance-assisted hydrogen bond (RAHB) skeleton of 1-AII, compared to other possible positional isomers, allows using it as a chelate by replacing the transferrable proton with metal ions and some metalloids. For example, the boron derivative (BH_2_-AII) of AII, was optimized using the B3LYP/6-31G(d,p) method. The structure shows that boron is bonded to the two nitrogen atoms and participates in a total of four bonds. For simplicity, the two hydrogen atoms (of BH_2_) were proposed but they may be replaced by halogen, oxygen, or nitrogen ligands. The TD-Cam-B3LYP/6-31G(d) calculation indicates that the first vertical HOMO-LUMO transition of this derivative occurs at a higher wavelength (compared to 1-AII and 2-AII tautomers) equal to 609 nm (∆E = 2.036 eV). This is a significant redshift with respect to the two tautomers. Among the other observations, the structure of BH_2_-AII is planar and the frontier orbitals distribute over the entire skeleton with a lower gap than the other two tautomers (Figure 7). 

## 4. Conclusions 

Being inspired by the outstanding pioneering achievements of scientists in the various related fields, and after carrying out the presented calculations, there are several points to highlight: 

1. To calculate the proton transfer energy barrier using the high-level MP4(SDTQ) theory to search many basis sets identified the popular 6-31G(d,p) basis set as a feasible description of atomic orbitals (reasonably balanced between accuracy and computational demands). Testing the MP4(SDTQ)/6-31G(d,p)//B3LYP/6-31G(d,p) protocol confirmed that it can produce satisfactory values compared to the heavier reference CCSD(T)/CBS and FP-CCSD(T) calculations and excellent consistency with the previously-recommended more-demanding protocols (CCSD(T) and QCISD(T), along with the D95(d,p) basis set). The identified MP4(SDTQ) protocol can be used to calculate the proton transfer energy barrier for a large number of known and unknown molecules. Additionally, it can be taken as a reference to evaluate the performance of other computational methods.

2. In addition to the importance of aromaticity, the MP4(SDTQ) calculations demonstrated that increasing the distance between the two nitrogen atoms (in the HNCCCN tautomerism segment) increases the value of the activation energy of the proton transfer process (N1 ↔ H ↔ N2), as expected. The MP4(SDTQ) calculations indicate that the barrier height of 4-amino-3-iminoindene is equal to 22 kcal/mol (compared to 9 kcal/mol in malonaldimine). This value means that 1-AII is the dominant structure, but generating the second tautomer to an appreciable amount using the suitable energy (stimuli-1) is kinetically possible. The small increase in the N1 ↔ N2 distance in 4-amino-3-iminoindene (compared to malonaldimine) proves the theory that the N1…H…N2 path length is a critical geometrical parameter in controlling the tautomerism process. 

3. Based on reference benchmark data, it appears that the TD-(CAM-B3LYP/6-31G(d)) calculation is suitable for estimating the first valence vertical excitation energy of these systems. These calculations illustrate that 2-AII is a better conductor than 1-AII at the molecular level, which fulfills the basic requirement of molecular switching and signaling applications. 

4. The effect of replacing the transferring proton by BH_2_ was examined. This substitution prevents the tautomerism phenomenon from happening and leads to a decrease in the energy difference between the frontier orbitals, which causes a significant redshift (244 nm) with respect to the more stable tautomer. The resulting structure is fully conjugated and planar.

5. In addition to this, the HOMO-LUMO gap of 4-amino-3-iminoindene tautomers and a boron derivative are similar to that of known molecular semiconductors (based on identical calculations). In addition to these two features, the relative position of the frontier orbitals may qualify the skeleton as an electron donor in conjunction with C60 or C70 receptors in organic solar cells.

**Figure 8:**
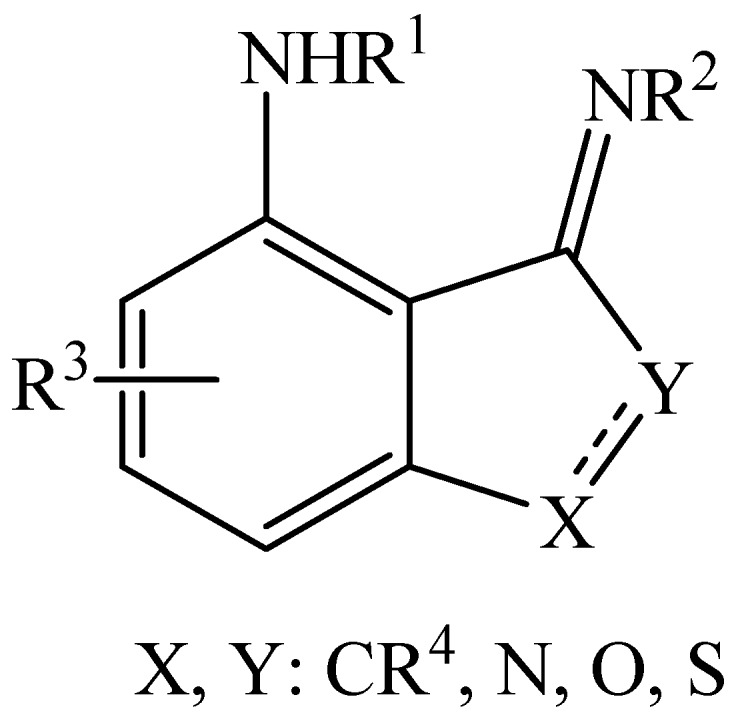
Suggested indene-like skeletons that may be suitable for molecular technology applications.

These results and insights encourage further investigations toward novel organic molecular materials. Figure 8 shows suggested similar molecular skeletons that may be investigated theoretically and experimentally. We are also interested in finding new factors that may affect tautomerism in interesting structures.

## Figures and Tables

**Table 1:** MP4(SDTQ)/(basis set)//B3LYP/6-31G(d,p) calculations based on malonaldehyde transition state. ∆Eb is the barrier height (in kcal/mol). |∆∆Eb| is the absolute difference with respect to the reference value (4.09 kcal/mol).

Basis Set	∆Eb	|∆∆Eb|	Basis Set	∆Eb	|∆∆Eb|
6-31G(d,p)	4.11	0.02	D95(d,p)	3.70	0.39
6-31++G(d,p)	3.87	0.22	EPR-II	3.05	1.04
6-311G(d,p)	3.81	0.28	SVP	3.83	0.26
6-311++G(d,p)	3.64	0.45	TZVP	3.32	0.77
cc-pVDZ	3.37	0.72			

**Table 2:** Performance of MP4(SDTQ)/6-31G(d,p)//B3LYP/6-31G(d,p) in estimating the proton transfer barrier of six malonaldehyde derivatives. |∆∆Eb| refers to the absolute deviation of the calculated value from the FP-CCSD(T) reference value.

	R = BH_2_	R = CN	R = NO_2_
2-R-Malonaldehyde	∆Eb	∆Eb	∆Eb
FP-CCSD(T)	2.62	3.56	3.34
MP4(SDTQ)/6-31G(d,p) (Average |∆∆Eb|= 0.19)	2.75 (0.13)	3.74 (0.18)	3.59 (0.25)
B3LYP/6-31G(d,p) (Average |∆∆Eb|= 1.32)	1.55 (1.07)	2.06 (1.50)	1.96 (1.38)
2-R-Malonamide			
FP-CCSD(T)	0.60	1.40	0.43
MP4(SDTQ)/6-31G(d,p) (Average |∆∆Eb|= 0.02)	0.58 (0.02)	1.40 (0.00)	0.46 (0.03)
B3LYP/6-31G(d,p) (Average |∆∆Eb|= 0.50)	0.21 (0.39)	0.61 (0.79)	0.12 (0.31)
1-AII → TS-AII		2-AII → TS-AII	
MP4(SDTQ)/6-31G(d,p)	22.41	MP4(SDTQ)/6-31G(d,p)	5.72
CCSD(T)/D95(d,p)	22.26	CCSD(T)/D95(d,p)	6.15
QCISD(T)/D95(d,p)	21.99	QCISD(T)/D95(d,p)	6.04
B3LYP/6-31G(d,p)	18.83	B3LYP/6-31G(d,p)	4.03

**Table 3:** The estimated vertical π → π* excitation energies (eV) and wavelength (nm) of 1-AII and 2-AII using the TD-(CAM-B3LYP/6-31G(d)) protocol in different media (ε is the dielectric constant). The values in parentheses were obtained using the TZVP basis set.

		1-AII		2-AII			
Medium	ε	∆E	λ	∆E	λ	|∆∆E|	∆λ
Vacuum	1	3.39 (3.37)	365 (368)	2.40 (2.39)	518 (518)	0.99 (0.98)	153 (150)
Acetonitrile	36.64	3.40	365	2.43	510	0.97	145
Water	78.39	3.40	364	2.43	510	0.97	146

**Table 4:** The TD-(CAM-B3LYP/6-31G(d)) frontier orbitals—the highest occupied molecular orbital (HOMO) and the lowest unoccupied molecular orbital (LUMO)—HOMO-LUMO gap (eV), the frontier orbitals energies (eV), and the first vertical excitation wavelength of AII tautomers and selected polyacenes.

Compound	Energy (eV)	HOMO	LUMO	λ (nm)
1-AII	3.39	–6.70	–0.65	365
2-AII	2.40	–6.07	–1.12	518
AII-BH_2_	2.04	–6.12	–1.44	609
Anthracene	3.63 (experimental: 3.60)	–6.44	–0.51	342
Tetracene	2.85 (experimental: 2.88)	–5.99	–1.03	435
Pentacene	2.31 (experimental: 2.37)	–5.67	–1.40	537
